# Characterization of x-type high-molecular-weight glutenin promoters (x-HGP) from different genomes in *Triticeae*

**DOI:** 10.1186/2193-1801-2-152

**Published:** 2013-04-10

**Authors:** Qian-Tao Jiang, Quan-Zhi Zhao, Xiu-Ying Wang, Chang-Shui Wang, Shan Zhao, Xue Cao, Xiu-Jin Lan, Zhen-Xiang Lu, You-Liang Zheng, Yu-Ming Wei

**Affiliations:** Triticeae Research Institute, Sichuan Agricultural University, Chengdu, Sichuan 611130 China; Lethbridge Research Centre, Agriculture and Agri-Food Canada, Lethbridge, T1J4B1 Canada; Key Laboratory of Southwestern Crop Germplasm Utilization, Ministry of Agriculture, Ya’an, Sichuan 625014 China

**Keywords:** Evolution analysis, Regulatory element, *Triticeae*, x-type high-molecular-weight glutenin promoter (x-HGP)

## Abstract

The sequences of x-type high-molecular-weight glutenin promoter (x-HGP) from 21 diploid *Triticeae* species were cloned and sequenced. The lengths of x-HGP varied from 897 to 955 bp, and there are 329 variable sites including 105 singleton sites and 224 polymorphic sites. Genetic distances of pairwise X-HGP sequences ranged from 0.30 to 16.40% within 21 species and four outgroup species of *Hordeum*. All five recognized regulatory elements emerged and showed higher conservation in the x-HGP of 21 *Triticeae* species. Most variations were distributed in the regions among or between regulatory elements. A 22 bp and 50 bp insertions which were the copy of adjacent region with minor change, were found in the x-HGP of *Ae. speltoides* and *Ps. Huashanica*, and could be regarded as genome specific indels. The phylogeny of media-joining network and neighbour-joining tree both supported the topology were composed of three sperate clusters. Especially, the cluster I comprising the x-HGP sequences of *Aegilops*, *Triticum*, *Henrardia*, *Agropyron* and *Taeniatherum* was highly supporting by both network and NJ tree. As conferring to higher level and temporal and spatial expression, x-HGP can used as the source of promoter for constructing transgenic plants which allow endosperm-specific expression of exogenous gene on higher level. In addition, the x-HGP has enough conservation and variation; so it should be valuable in phylogenetic analyses of *Triticeae* family members.

## Introduction

In wheat and its relatives, high-molecular-weight glutenin subunits (HMW-GSs) are one of the most important storage proteins in seed endosperm as their significant effects on wheat processing quality ( Lawrence and Shepherd 1980 ; Payne 1987 ; Shewry et al. 1992 ). HMW-GSs are critical in determining wheat gluten and dough elasticity which promote the formation of the larger glutenin polymer (Shewry et al. 1995 ). The genes encoding for HMW-GSs are designated as *Glu-1* loci locating on the long arms of the Group 1 chromosomes in bread wheat. Each *Glu-1* locus consists of 2 tightly linked genes encoding an x-type subunit with a larger molecular weight and a y-type subunit with a smaller one, respectively ( Payne 1987 ). Up to now, a lot of studies have been conducted in identifications and function analysis of HMW-GS genes from wheat and its wild relatives ( Anderson and Greene 1989 ; Forde et al. 1985 ; Halford et al. 1987 ; Jiang et al. 2012a ; Jiang et al. 2012b ; Jiang et al. 2009 ; Liu et al. 2003, 2007, 2008, 2010 ; Sugiyama et al. 1985 ; Thompson et al. 1985 ; Wan et al. 2005 ).

HMW-GS genes and other seed protein encoding genes share similar expression pattern of tissue-specific and developmental regulation even though they have different regulatory elements (Lamacchia et al. 2001 ; Shewry and Halford 2002 ). Previous studies indicated that high-molecular-weight glutenin promoter (HGP) contains five recognized regulatory elements, they are transcription start site, TATA box, complete HMW enhancer, partial HMW enhancer, the prolamin box like element which is composed of two relatively conserved motifs: the endosperm motif (E motif) and the GCN4-like motif (N motif)(Hammond-Kosack et al. 1993 ; Müller and Knudsen 1993 ). Based on the regulation of these elements, the encoding genes of HMW-GS exhibit a higher expression level than those of other seed storage proteins (Lamacchia et al. 2001 ). The grasses of the *Triticeae* tribe include huge number of wheat and its relatives, which has been widely researched as genetic resource for wheat quality improvement programs. For example, previous reports revealed that wild species has abundant HMW-GS variants which confers to different structural feature and expression level from those of common wheat (Jiang et al. 2012a ; Liu et al. 2010 ; Wan et al. 2002 ; 2005 ).

In previous study, we have characterized y-type HGP and its cis regulatory elements from 25 *Triticeae* species (Jiang et al. 2010 ). In this study, we further reported the characterization of x-type high-molecular-weight glutenin promoter (x-HGP) in 21 diploid *Triticeae* species. The objective of this study is to investigate molecular information for x-HGP in 21 diploid species of *Triticeae*, and characterize regulatory elements, and explore phylogenetic relationship among x-HGP of different species of *Triticeae*.

## Materials and methods

### Plant materials

Twenty-one diploid species of *Triticeae* were investigated in this study, and four *Hordeum* species were used as outgroup (Table 1). The accessions with PI numbers were kindly provided by USDA-ARS (http://www.ars-grin.gov/npgs/). The accessions with AS numbers were deposited at *Triticeae* Research Institute, Sichuan Agricultural University, China.

**Table 1:** **The 25 diploid species of*Triticeae*used in this study**

***Accession***	***Taxon***	***Abbreviation***	***Genome***	***Origin***	***GenBank***	***References***
PI428311	*Triticum urartu* Tumanian ex Gandilyan	TRUR	A^u^	Beqaa, Lebanon	KC478921	This study
PI428007	*Triticum monococcum* L. subsp.*aegilopoides* (Link) Thell.	TRBO	A^m^	Arbil, Iraq	KC478922	This study
CIae 70	*Aegilops bicornis* (Forsskal) Jaub. & Spach	AEBI	S^b^	Unknown	KC478923	This study
PI 604122	*Aegilops longissima* (Schweinf. & Muschl.) Á. Löve.	AELO	S^l^	Central, Israel	KC478924	This study
PI599149	*Aegilops searsii* (Feldman & Kislev ex Hammer) Á. Löve	AESE	S^s^	Southern, Israel	KC478925	This study
PI 584388	*Aegilops sharonensis* (Eig) Á. Löve.	AESH	S^sh^	Haifa, Israel	KC478926	This study
PI560531	*Aegilops speltoides* (Tausch) Á.Löve	AESP	S	Turkey	KC478927	This study
PI603230	*Aegilops tauschii* (Coss) Á. Löve.	AETA	D	Azerbaijan	KC478928	This study
PI531711	*Thinopyrum bessarabicum* (Savul. & Rayss) A. Love	THBE	E^b^	Ukraine	KC478929	This study
PI 578683	*Thinopyrum elongatum* (Host) D. R. Dewey	THEL	E^e^	Nebraska	KC478930	This study
PI219966	*Eremopyrum bonaepartis* (Spreng.) Nevski	ERBO	F	Afghanistan	KC478931	This study
PI276970	*Aegilops comosa*	AECO	M	Greece	KC478932	This study
PI531823	*Psathyrostachys huashanica* Keng	PSHU	Ns	Shanxi, China	KC478933	This study
PI577112	*Henrardia persica* (Boiss.) C. E. Hubb	HEPE	O	Turkey	KC478934	This study
PI277352	*Agropyron cristatum* (L.) Grossh	AGCR	P	Former Soviet Union	KC478935	This study
PI283983	*Secale sylvestre*	SESY	R	Former Soviet Union	KC478936	This study
PI205222	*Secale strictum*	SEST	R	Eskisehir, Turkey	KC478937	This study
PI168199	*Secale cereale*	SECE	R	Isparta, Turkey	KC478938	This study
AS136	*Aegilops uniaristata* Vis	AEUN	N	Unknown	KC478939	This study
PI220590	*Taeniatherum caput-medusae*	TACA	Ta	Afghanistan	KC478940	This study
AS2	*Aegilops umbellulata* Zhuk	AEUM	U	Unknown	KC478941	This study
PI 499645	*Hordeum bogdanii* Wilensky	HOBO	H	Xinjiang, China	EU074248	Jiang et al. 2010
PI383667	*Hordeum brevisubulatum* Bothmer	HOBR	H	Erzurum, Turkey	EU074247	Jiang et al. 2010
PI401357	*Hordeum bulbosum*	HOBU	I	Iran	EU074249	Jiang et al. 2010
PI466482	*Hordeum spontaneaum* (K. Koch) Thell	HOSP	I	Israel	EU074250	Jiang et al. 2010

### Isolation and sequencing of x-HGP from *Triticeae* species

**Figure 1:**
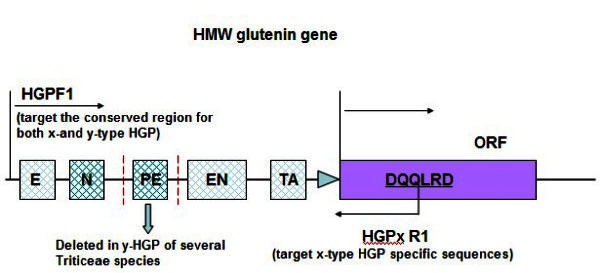
**Schematic structure of HMW glutenin gene promoter and the strategy of cloning.** The regulatory elements were indicated by boxes, E: E motif, N: N motif, PE: partial HMW enhancer, EN: complete HMW enhancer, TA: TATA box. The specific primers (HGPF and HGPxR) for amplifying x-type HMW glutenin gene promoter and their target region are marked. The deletion of regulatory element *partial enhancer* in y-type HMW glutenin gene promoter of some species is also indicated by broken lines.

Genomic DNA was extracted from the leaves of two-week-old single plant by using CTAB extraction method ( Murray and Thompson 1980 ). To design x-type specific primers, we aligned the published sequences of HMW glutenin genes *1Ax1* (GenBank: *X61009*), 1Ax2* (GenBank: *M22208*), 1Bx7 (GenBank: *X13927*), 1Bx17 (GenBank: *JC2099*), 1Dx2 (GenBank: *X03346*), 1Dx5 (GenBank: *X12928*), 1Ay (GenBank: *X03042*) 1By9 (GenBank: *X61026*), 1Dy10 (GenBank: *X12929*), and 1Dy12 (GenBank: *X03041*). According to the results of alignment, a pair of primers (HGPF and HGPxR) was designed to specifically amplify x-HGP. The HGPF1 primer (5^′^-AGGGAAAGACAATGGACATG -3^′^) was designed from the sequence which was highly conserved in the 5^′^ upstream regions of both x- type and y- type HGP, whereas the HGPxR1 primer (5^′^- GTCTCGGAGC/TTGC/TTGGTC-3^′^) was targeted to the sequence coding for six amino acid residues (DQQLRD) which appear only in the N-terminal domain of x-type HMW-GSs (Figure 1). The amplification profile was 94°C for 5 min, followed by 35 cycles of 94°C for 45 sec, 60°C for 1 min, and 72°C for 2 min 30 sec, and a final extension step at 72°C for 10 min. High-fidelity LA Taq polymerase (Takara, Dalian, China) was used in the PCR reactions to avoid introducing errors into the sequence. The amplified products were separated by 1.0% agarose gels. Purified PCR products were then ligated into pMD19-T vector (Takara, Dalian China). The amplified products were purified and ligated into the pMD19-T vector (TaKaRa, Dalian, China). The cloned fragments were sequenced in both directions by a commercial company (Invitrogen, Shanghai, China). The sequencing results of three independent clones at least were used to determine the final nucleotide sequence of each species. All the DNA sequences have been deposited into the NCBI database with the GenBank accession numbers from KC478921 to KC478941 (Table 1).

### Data analyses

The sequence prediction was performed by DNAman software package (Version 5. 2. 10; Lynnon Biosoft). The sequence alignment was carried out with Clustal W Version 1.83(Thompson et al. 1994 ). The alignment was further improved by visual examination and manual adjustment. The y-HGP sequences of four *Hordeum* species were used as outgroup. The genetic distance was calculated by using the software Mega (Version 4.02) with the parameters, nucleotide model: Kimura 2-parameter, and substitution: Transitions + Transversions (Tamura et al. 2007 ). To enhance the comparison between wheat and its relatives, the sites with informative variations were used to construct media-joining network in program Network 4.6.1.1 (http://www.fluxus-engineering.com) with the following parameters of weights = 10, epsilon = 0 and the transversions /transitions ratio was set to 3:1 ( Allaby and Brown 2001 ; Bandelt et al. 1999 ). The media-joining network was calculated under the parameters of weights = 10, epsilon = 0 and the transversions /transitions ratio was set to 3:1 ( Allaby and Brown 2001 ). The neighbour-joining (NJ) tree was constructed to estimate the possibility of phylogenetic clade, under the substitute model of Maximum Composite Likelihood; gaps were treated as missing data. To estimate the topological robustness, the bootstrap values were calculated based on 1000 replications.

## Results

### Sequence variation and structural characteristics of x-HGP

**Figure 2:**
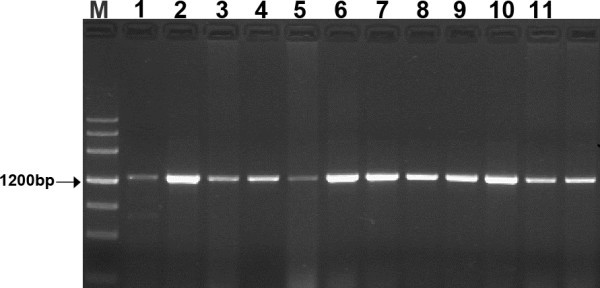
**PCR amplification of x-HGP from partial of 21*Triticeae*species.** Line 1–12: *T. urartu*, *T. monococcum* L. subsp.*aegilopoides*, *Ae. bicornis*, *Ae. longissima*, *Ae. sharonensis*, *Ae. speltoides*, *Ae. tauschii*, *Ps. huashanica*, *Se. cereale*, *Ta. caput-medusae*, *Th. Elongatum* and *Th. Elongatum*; M is DNA marker.

**Figure 3:**
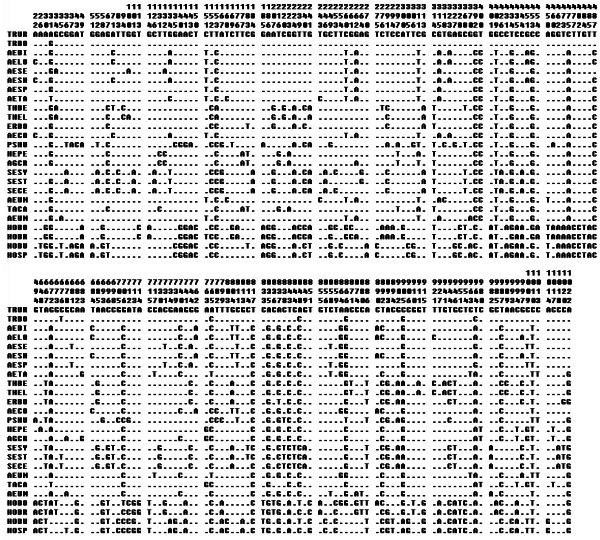
**The nucleotide variations in the sequences of x-HGP from 21 Tritceae species and y-HGP of*Hordeu*m.** The number at the top of figure indicated the variation position. See Table 1 for species abbreviations.

**Figure 4:**
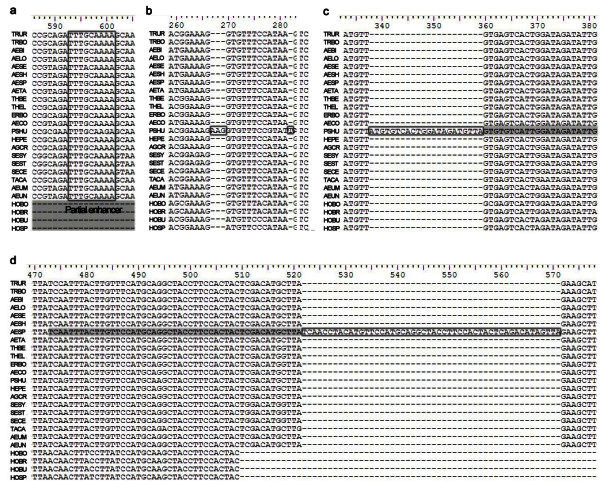
**Multiple sequence alignment of x-HGP of 21*Triticeae*diploid species and four species of*Hordeum*as outgroup.** The species-specific indels were indicated by boxes, **a**, partial HMW enhancer appears in all x-HGP of all 21 species, but deleted in those of four *Hordeum*; **b** and **c**, **d** represent unique indels in *Psathyrostachys* and *Aegilops*. The inserted fragments in *Psathyrostachys* and *Aegilops* are duplication of adjacent region.

In genomic PCR, there is only one fragment of approximately 1200 bp were amplified in each of 21 diploid *Triticeae* species by using the x-HGP specific primers HGPF1 + HGPxR1 (Figure 2). The PCR fragments were cloned and sequenced. And the final x-HGP sequence of each species was assembled by at least three independent clones. The results of sequencing showed that the lengths of x-HGP from which the sequences encoding signal peptide and partial N-terminal varied from 897 to 955 bp. The x-HGP sequences were different from each other by substitutions, insertions and deletions of single or more nucleotides. Although there is difference in DNA sequences, the x-HGP exhibit higher conservation among different genomes of *Triticeae*. For all the sequences, there are 329 variable sites including 105 singleton sites and 224 polymorphic sites, of which 192sites were informative (Figure 3). According to the sequence characteristics and location of identified elements, we characterized all five recognized regulatory elements and summarized their variations in Table 2. The sequences of these regulatory elements showed higher conservation, for example, the N motif share perfect identical sequences among all 21 species of *Triticeae*. The sequence variations of rest of elements only resulted from single or few base substitutions except for single base deletion in the motif Enhancer of *Aegilops speltoides* and *Thinopyrum bessarabicum*. The insertions and deletions (Indels) was the main cause of length variation of x-HGP among 21 species. Most variations distributed in the regions among or between regulatory elements. A few genome specific indels were also characterized in *Ae. speltoides* and *Psathyrostachys huashanica* (Figure 4a, b). A 22 bp and 50 bp insertions were found in the x-HGP of *Ae. speltoides* and *Ps. huashanica*. These inserted fragments are the copies of adjacent region, of which the duplication has some mutation involving single base pair in *Ps. huashanica* (Figure 4c, d).

**Table 2:** **Sequence variations of element of x-HGP from 21 species in*Triticeae*and outgroups, y-HGP from four species of*Hordeum***

***Species***	***E motif***	***N motif***	***Partial Enhancer***	***Enhancer***	***TATA box***	***Start***
	***(TGTAAAGT)***	***(TGAGTCAT)***	***(TTTGCAAA)***	***(GTTTTGCAAAGCTCCAATTGCTCCTTGCTT ATCCAGCT)***	***(CTATAAAAG)***	***(TTATCA)***
TRUR	TGTAAATC	TGAGTCAT	TTTGCAAA	GTTTTACAAAGCTCCAATTGCTCCTTGCTTATCCAGCT	CTATAAAAG	TCTTCA
TRBO	TGTAAATC	TGAGTCAT	TTTGCAAA	GTTTTGCAAAGCTCCAATTGCTCCTTGCTTATCCAGCT	CTATAAAAG	TCCTCA
AEBI	TGTAAATC	TGAGTCAT	TTTGCAAA	GTTTTGCAAAGCTCCAATTGCTCCTTTCTTATCTAGCT	CTATAAAAG	TCATCA
AELO	TGTAAATC	TGAGTCAT	TTTGCAAA	GTTTTGCAAAGCTCCAATTGCTCCTTTCTTATCTAGCT	CTATAAAAG	TCATCA
AESE	TGTAAATC	TGAGTCAT	TTTACAAA	GTTTTGCAAAGCTCCAATTGCTCCGTGCTTATCTAGCT	CTATAAAAG	TCGTCA
AESH	TGTAAATC	TGAGTCAT	TTTGCAAA	GTTTTGCAAAGCTCCAATTGCTCCTTTCTTATCTAGCT	CTATAAAAG	TCATCA
AESP	TGTAAATC	TGAGTCAT	TTTGCAAA	GTTTTGCAA-GCTCCAATTGCTCCTTGCTTATCTAGCT	CTATAAAAG	TCGTCA
AETA	TGTAAATC	TGAGTCAT	TTTGCAAA	GTTTTGCAAAGCTCCAATTGCTCCTTGCTTATCCAGCT	CTATAAAAG	TTATCA
THBE	TGTAAATC	TGAGTCAT	TTTGCAAA	-TTTTGCAAAGCTCCAATTGCTCCTTACTTATCCAGCT	CTATAAAAA	TCATCA
THEL	TGTAAATC	TGAGTCAT	TTTGCAAA	GTTTTGCAAAGCTCCAATTGCTCCTTACTTATCCAGCT	CTATAAAAA	TCATCA
ERBO	TGTAAATC	TGAGTCAT	TTTGCAAA	GTTTTGCAAAGCTCCAATTGCTCCTTACTTATCCAGCT	CTATAAAAG	TCATCA
AECO	TGTAAATC	TGAGTCAT	TTTGCAAA	GTTTCGCAAAGCTCCAATTGCTCCTTTCTTATCTAGCT	CTATAAAAG	TCATCA
PSHU	TGTAAGTT	TGAGTCAT	TTTGCAAG	GTTTCGCAAAGCTCCAATTGCCCCTTGCTTATCTAGCT	CTATAAAAG	TCATCA
HEPE	TGTAAATC	TGAGTCAT	TTTGCAAA	GTTTTGCAAAGCTCCAATTGCTCCTTGCTTATTCAGCT	CTATAAAAG	TCATCA
AGCR	TGTAAATC	TGAGTCAT	TTTGCAAA	GTTTTGCAAAGCTCCAATTGCTCCTTGCTTATCCAGCT	CTATAAAAG	TCATCA
SESY	TGTAAGTC	TGAGTCAT	TTTGCAAA	GTTTTGCAAAGCTCCAATTGCTCCTTACTTATCCAGTT	CTATAAAAG	TCATCA
SEST	TGTAAGTC	TGAGTCAT	TTTGCAAA	GTTTTGCAAAGCTCCAATTGCTCCTTACTTATCCAGTT	CTATAAAAG	TCATCA
SECE	TGTAAGTC	TGAGTCAT	TTTGCAAA	GTTTTGCAAAGCTCCAATTGCTCCTTACTTATCCAGCT	CTATAAAAG	TCATCA
AEUN	TGTAAATC	TGAGTCAT	TTTGCAAA	GTTTTGCAAAGCTCCAATTGTTCCTTGCTTATCCAGCT	CTATAAAAG	TCATCA
TACA	TGTAAATC	TGAGTCAT	TTTGCAAA	GTCTTGCAAAGCTCCAATTGCTCCTTGCTTATCCAGCT	CTATAAAAG	TCATCA
AEUM	TGTAAACC	TGAGTCAT	TTTGCAAA	GTTTTGCAAAGCTCCAATTGCTCCTTGCTTATCCAGCT	CTATAAAAG	TTATCA
HOBO	TGTAAATG	TGAGTCAT	deleted	GTTTTGCAAAGCTCCAATTGCACCTTGCTTATCCAGCT	CTATAAAAG	TCATCA
HOBR	TGTAAATG	TGAGTCAT	deleted	GTTTTGCAAAGCTCCAATTGCACCTTGCTTATCCAGCT	CTATAAAAG	TCATCA
HOBU	TGTAAATC	TGAGTCAT	deleted	ATTTTGCAAAGCTCCAATTGCACCTCGCTTATCCAACT	CTATAAAAG	TCATCA
HOSP	TGTAAATC	TGAGTCAT	deleted	GTTTTGCAAAGCTCCAATTGCACCTCGCTTATCCAACT	CTATAAAAG	TCATCA

The positions of variation were underlined in the corresponding loci of consensus sequences.

The number of transitions and transversions are listed in Table 3. The transitions/transversions ratios of the x-HGP sequences varied from 0 to 21, showing the nucleotide substitution rates were unequal within *Triticeae*. Genetic distances of pairwise X-HGP sequences ranged from 0.30 to 16.40% within 21 species and four outgroup species of *Hordeum* (Table 3). The pairwise x-HGP divergence values were low and were coincided to higher conservation of x-HGP sequences in different genomes of *Triticeae*.

**Table 3:** **Pairwise comparisons of nucleotide substitutions and genetic distances of x-HGP sequences of 21*Triticeae*species and y-HGP sequences of four*Hordeum*species**

	TACP	AESP	SESY	AEBI	AECO	AELO	AESH	AETA	AESE	AEUM	AEUN	AGCR	THBE	THEL	ERBO	HOBO	HOBR	HOBU	HOSP	HEPE	PSHU	SEST	SECE	TRBO	TRUR
TACP		4.70	8.60	5.50	5.10	5.50	5.30	5.10	5.80	5.50	5.00	1.70	7.20	6.40	5.00	15.70	14.60	13.50	14.30	2.10	11.10	8.50	8.30	5.70	5.80
AESP	34/9		8.40	2.40	2.10	2.40	2.20	2.30	1.00	3.00	2.50	3.80	6.90	6.10	5.00	14.60	13.40	13.70	14.20	5.00	10.70	8.20	8.10	4.60	4.80
SESY	51/22	53/17		8.90	8.50	8.80	8.50	8.50	9.50	8.90	8.50	8.50	5.30	5.00	3.90	15.80	14.90	14.80	15.30	9.10	11.20	0.40	0.50	9.20	9.00
AEBI	34/15	14/5	51/22		0.90	1.00	0.80	3.10	3.50	3.90	3.10	4.60	7.50	6.80	5.70	15.20	14.30	14.80	15.30	5.80	11.40	8.80	8.60	5.70	5.90
AECO	31/13	12/5	50/20	4/3		0.60	0.40	2.70	3.10	3.50	2.70	4.20	7.10	6.40	5.30	14.70	13.80	14.30	14.80	5.40	10.60	8.30	8.20	5.30	5.50
AELO	34/14	15/4	51/21	5/3	5/0		0.30	3.10	3.50	3.90	2.80	4.60	7.10	6.40	5.50	15.20	14.30	14.80	15.30	5.80	11.40	8.60	8.50	5.70	5.90
AESH	32/14	13/4	49/20	3/3	3/0	2/0		2.80	3.20	3.60	2.60	4.30	7.10	6.40	5.30	14.90	14.00	14.50	14.90	5.50	11.10	8.30	8.20	5.40	5.70
AETA	34/14	18/3	55/18	18/8	16/7	19/7	17/7		3.40	3.20	1.10	3.90	7.20	6.50	5.40	15.00	13.80	14.30	14.80	5.40	10.90	8.50	8.40	5.50	5.50
AESE	40/11	7/2	60/19	20/8	19/6	22/6	14/2	20/5		4.10	3.50	4.70	7.80	6.60	6.10	15.70	14.60	15.00	15.50	6.00	11.90	9.40	9.20	5.40	5.70
AEUM	36/17	18/5	54/22	23/10	21/9	24/9	22/9	22/8	28/7		3.40	4.30	7.80	6.90	5.80	15.20	13.80	14.30	14.60	5.80	11.70	8.80	8.60	6.00	6.20
AEUN	36/12	21/1	56/18	22/6	19/5	20/5	18/5	6/4	28/3	30/5		4.00	7.10	6.20	5.10	14.60	13.40	13.90	14.30	5.30	10.50	8.40	8.20	5.40	5.50
AGCR	13/3	27/8	53/21	29/12	17/11	30/11	28/11	26/10	33/10	30/12	30/8		7.10	6.20	4.80	15.30	13.90	13.40	14.00	1.70	10.70	8.40	8.20	5.80	5.90
THBE	43/21	34/15	31/15	39/19	41/19	42/18	43/18	43/19	47/18	46/20	44/17	42/20		1.10	2.70	14.90	13.60	13.70	14.10	7.60	10.60	5.10	5.00	7.90	8.00
THEL	37/22	37/15	31/15	37/21	35/19	35/20	34/20	39/19	39/19	39/21	40/17	37/20	7/2		2.20	14.70	13.50	13.10	13.50	6.80	10.30	4.90	4.70	7.00	7.20
ERBO	29/15	31/10	26/9	32/15	30/14	32/14	30/14	34/13	39/12	36/15	35/11	30/14	17/7	12/8		13.50	12.20	11.40	12.10	5.40	9.20	3.80	3.60	6.10	6.20
HOBO	67/47	67/40	71/47	68/43	66/42	69/42	67/42	68/42	70/43	70/41	69/39	69/45	63/47	63/48	57/44		3.90	9.60	10.20	15.80	16.40	16.00	15.50	15.80	16.00
HOBR	64/43	62/35	71/45	63/41	61/40	64/40	67/40	63/39	68/35	64/36	64/35	63/41	59/43	58/44	53/40	23/8		8.70	9.00	14.70	15.20	14.70	14.60	15.00	15.00
HOBU	65/33	70/31	76/36	71/34	69/34	74/33	70/34	73/33	76/31	71/32	66/27	68/33	66/37	64/37	56/33	49/29	45/28		1.30	13.90	14.30	14.70	14.50	14.50	14.10
HOSP	63/37	68/34	83/45	70/39	67/38	70/38	68/37	70/37	74/36	68/37	71/33	60/32	67/40	61/41	55/37	47/33	40/31	8/4		14.60	14.80	15.10	14.90	14.90	14.70
HEPE	16/1	35/10	55/22	36/16	34/14	37/14	35/14	36/13	41/12	39/15	36/11	12/3	46/20	40/21	33/15	69/43	66/42	68/34	37/38		11.40	8.90	8.80	6.40	6.50
PSHU	61/33	60/30	63/39	59/34	56/34	61/32	59/34	60/33	67/32	63/35	60/31	59/31	58/37	56/37	51/32	68/53	64/49	71/39	69/43	64/30		11.10	10.90	12.40	12.60
SEST	53/21	52/17	3/0	51/22	49/20	51/21	49/21	55/18	59/19	54/21	52/18	53/20	33/15	30/15	24/9	72/47	67/43	75/36	73/39	55/22	62/39		0.40	8.80	9.20
SECE	52/22	51/17	4/0	49/23	48/21	50/21	48/21	54/18	58/19	53/22	53/18	51/20	31/11	29/15	24/9	69/28	66/43	74/35	70/40	54/22	60/39	3/0		8.60	9.00
TRBO	34/18	29/10	52/25	32/15	30/14	33/14	31/14	35/13	33/13	37/15	37/10	35/17	44/24	38/24	33/17	69/47	67/43	68/37	69/37	39/18	66/26	50/25	49/25		1.70
TRUR	32/20	27/12	46/27	31/17	24/16	32/16	30/16	33/15	33/13	36/17	35/13	33/19	42/21	36/24	32/20	68/49	66/44	66/39	64/42	37/20	65/39	49/27	49/32	10/4	

Note: Percentage of sequence divergence using genetic distance is shown in the upper diagonal. Direct counts of transitions/transversions are shown in the lower diagonal. Species abbreviations are listed in Table 1.

### Phylogenetic analyses

**Figure 5:**
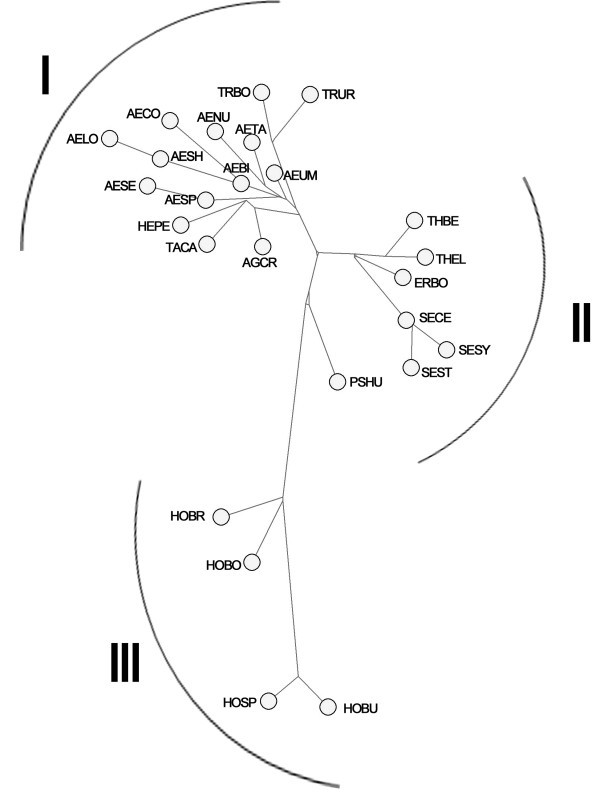
**The media-joining network derived from the x-HGP sequences from 21 diploid species of*Triticeae*and four y-HPG sequences of*Hordeum.*** The x-HGP of all *Aegilops* formed the biggest subcluster around which two minor clade comprising *Triticum*, and *He. persica*, *Ag. cristatum* and *Ta. caput-medusae* emerged at the top of network. The topology was cluster into three main separate groups with placing PSHU aside the group II.

The media-joining network analysis for 21 x-HGP and four y-HGP from different *Triticeae* genomes showed that the formed phylogeny is composed of three separate clusters (Figure 5). In the cluster I, the HGP of *Ae. bicornis*, *Ae. comosa*, *Ae. longissima*, *Ae. searsii*, *Ae. sharonensis*, *Ae. speltoides*, *Ae. tauschii*, *Ae. uniaristata*, *Ae. umbellulata*, *T. urartu*, *T. boeoticum*, *Henrardia persica*, *Agropyron cristatum* and *Taeniatherum caput-medusae* were inclued. The x-HGP of all *Aegilops* formed the biggest subcluster around which two minor clade comprising *Triticum*, and *Henrardia persica*, *Agropyron cristatum* and *Taeniatherum caput-medusae* emerged (at the top of Figure 5). The second cluster is composed of *Secale sylvestre*, *Se. strictum*, *Se. cereale*, *Th. bessarabicum*, *Th. elongata* and *Eremopyrum bonaepartis*, and the x-HGP of *Secale* and *Thinopyrum* species formed a separate clade, respectively (in the middle of Figure 5). For the third cluster, it’s composed of y-HPG of four *Hordeum*, and this cluster was further divided into two clades, one includes species of *H. bogdanii* and *H. brevisubulatum* with genome H, the other contains *H. bulbosum* and *H. spontaneaum* with the genome I (at the bottom of Figure 5).

**Figure 6:**
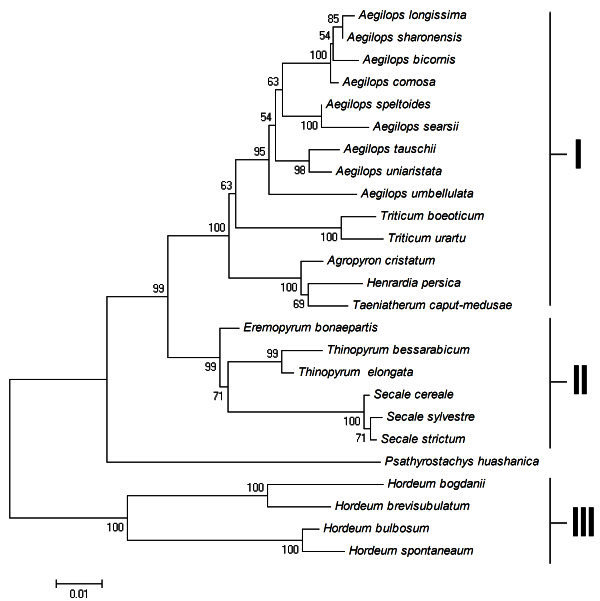
**The neighbor-joining (NJ) tree derived from x-HGP sequences from 21 diploid species of*Triticeae.*** The NJ tree was constructed by using the substitute model of Maximum Composite Likelihood. The bootstrap values were calculated based on 1000 replications to estimate the topological robustness.

The resulted neighbour-joining (NJ) trees showed highly identical topology to media-joining network (Figure 6), strongly supporting placement of three clusters. In addition, these clusters are also supported by high bootstrap values, indicating that strong statistic support for the reliability of phylogeny.

## Discussions

As a key factor in wheat quality, HMW-GS is one of most important storage protein in wheat seed. Although they only hold about 10% of seed storage proteins, the allelic variation in HMW-GS compositions has been reported to account for up to 70% of the variation in bread making quality among European wheats (Halford et al. 1992 ; Payne 1987 ; Wan et al. 2002 ). Apart from allelic variations in HMW-GS genes, variation in promoter regions of these genes also very useful to distinguish between the genes and gives better evolutionary studies among Triticeae family members (Anderson et al. 1998 ). Two ways were adopted to ensure the accuracy of results. Firstly, the high fidelity polymerase was used to ensure to avoid the potential mistakes introduced into the amplified fragments in genomic PCR. Secondly, to exclude probable errors in sequencing, each nucleotide sequence of x-HGP was determined by using sequencing results of multiple independent clones. Therefore, the molecular information we generated for x-HGP is reliable and effective for exploring structural variation and evolution among different species of *Triticeae*.

### The structure variations and evolution of x-HGP

HMW-GS genes are different from other prolamin genes at a higher expressional level. Under the regulation of high-molecular-weight glutenin promoter (HGP), single active HMW-GS gene encodes a subunit accounting for approximate 2% of total protein in mature wheat seed (Halford et al. 1992 ). This indicates that HGP confer to higher expression to HMW-GS gene. In our previous study of y-HGP from *Triticeae*, we found the regulatory element Partial Enhancer was deleted in eight species of *T*. *urartu*, *T*. *boeotum*, *Ae*. *umbellulata*, *Ae*. *uniaristata*, *H*. *bulbosum*, *H*. *spontaneum*, *H*. *bogdanii* and *H*. *brevisubulatum* (Jiang et al. 2010 ). In this study, the Partial Enhancer appeared in x-HGP of all 21 species of *Triticeae* (Figure 4a). The obvious variations were two large insertions in spacer region between regulatory elements within x-HGP of *Ae. speltoides* and *Ps. huashanica*. And the inserted fragments are the copy of adjacent region with minor variations (Figure 4c, d). The 85 bp-fragment deletion in the promoter region of inactive HMW subunit gene *1Ay* had been regarded as the possible reason for silencing of this allele (Halford et al. 1989 ). Our previous study revealed that this fragment has also been deleted in the active *1Ay* genes (Jiang et al. 2009 ). Previous study indicated that the 185 bp insertion in 1Bx7 promoter do not affect the expressions of HMW-GS genes (Harberd et al. 1987 ). We found that HMW-GS genes were usually disrupted by the variations in ORFs, such as premature stop codons, large transposon-like elements, etc. (Harberd et al. 1987 ; Jiang et al. 2012b ; Jiang et al. 2009 ). Therefore, the 22 bp and 50 bp insertion located in the regions between elements may not affect the expressions of HMW-GS genes. We conclude that this high conservation of regulatory elements is coincided to keep the tissue specificity and expression level of HMW-GS gene.

### Phylogenetic analysis of x-HGP among different species of *Triticeae*

There is only one D-hordein gene in *Hordeum*, which was orthologous of HMW-GS wheat and showing homology to y-type HMW-GS (Gu et al. 2003 ). Sequence analysis indicated that the y-HGP sequences of *Hordeum* shared homology in composition of regulatory elements with that of x-HGP of 21 *Triticeae* species, and have enough variations (supported by average distance of 12.60 among *Hordeum* and other species) among them. Therefore, using the sequences of *Hordeum* y-HGP as outgroups was suitable in phylogenetic analysis. The resulted media-joining network and neighbour-joining tree both supported the topology were composed of three sperate clusters. The cluster I, the biggest group, was highly supporting by both network and NJ tree, mainly including the x-HGP of all nine species of *Aegilops*, two species of *Triticum*, then *He. persica*, *Ag. cristatum* and *Ta. caput-medusae* were place aside. This group is high similar to the ones, *Aegilops-Triticum* complex and the Mediterranean clade identified in y-HGP and ITS phlylogenetic analysis, respectively(Hsiao et al. 1995 ; Jiang et al. 2010 ). It could be explained by their similar distribution in Mediterranean and neighbor regions. The x-HGP of *Thinopyrum*, *Secale* and *Hordeum* were clustered as subcluster according to their same genome. The genus *Hordeum* contains about 31 diploid and polyploid species, and four sections were determined by morphological characters (von Bothmer et al. 1995 ). Previous phylogenetic analysis by using ITS sequences has revealed four major clades that coincide with the four genome designations in *Hordeum* (Blattner, 2004, 2006 ). In our study, the x-HGP phylogenetic analysis also support the similar clades in *Hordeum*, respectively. Our results confirmed that x-HGP, like y-HGP and ITS, all can generate a good resolution to phylogenetic relationships within *Triticeae*.

In conclusion, according to the results of x-HGP sequences from 21 species in *Triticeae*, we conclude the x-HGP would be beneficial: 1) to drive exogenous gene to expresson on temporal and spatial pattern; 2) to serve as a valuable candidate in phylogenetic analyses of *Triticeae*.
